# Sources of Environmental Reinforcement and Engagement in Health Risk Behaviors Among a General Population Sample of US Adults

**DOI:** 10.3390/ijerph21111390

**Published:** 2024-10-22

**Authors:** Alexa M. L’Insalata, Jeffrey M. Girard, Tera L. Fazzino

**Affiliations:** 1 Department of Psychology, University of Kansas, Lawrence, KS 66045, USA; alinsalata@ku.edu (A.M.L.); jmgirard@ku.edu (J.M.G.); 2 Cofrin Logan Center for Addiction Research and Treatment, University of Kansas, Lawrence, KS 66045, USA

**Keywords:** behavioral choice, binge eating, alcohol, nicotine, exploratory structural equation modeling

## Abstract

Research supports the premise that greater substance use is associated with fewer sources of environmental reinforcement. However, it remains unclear whether types of environmental reinforcement (e.g., social or work) may differentially influence use. This study tested the association between types of environmental reinforcement and engagement in multiple health risk behaviors (alcohol use, binge eating, and nicotine use). Cross-sectional data were collected from a general population sample of US adults (*N* = 596). The Pleasant Events Schedule (PES) was used to measure sources of reinforcement. Exploratory structural equation modeling (ESEM) characterized different areas of environmental reinforcement and correlations with alcohol consumption, binge eating, and nicotine use. A four-factor structure of the PES demonstrated a conceptually cohesive model with acceptable fit and partial strict invariance. Social-related reinforcement was positively associated with alcohol consumption (*β* = 0.30, *p* < 0.001) and binge eating (*β* = 0.26, *p* < 0.001). Work/school-related reinforcement was negatively associated with binge eating (*β* = −0.14, *p* = 0.006). No areas of reinforcement were significantly associated with nicotine use (*p* values = 0.069 to 0.755). Social-related activities may be associated with engagement in multiple health risk behaviors (more binge eating and alcohol use), whereas work/school-related activities may be preventative against binge eating. Understanding these relationships can inform prevention efforts targeting health risk behaviors.

## 1. Introduction

Behavioral choice theories posit that the degree to which individuals seek out reinforcement from substance use (e.g., alcohol and nicotine) is related to the degree to which individuals seek out alternative sources of reinforcement from their environment (see reviews: [1,2]). Through this lens, substance use can be viewed as a behavioral process that is influenced by the availability of alternative sources of reinforcement in an individual’s environment. Alternative sources of reinforcement can be obtained from various contexts and sources, such as social activities (e.g., spending time with friends) and work (e.g., engaging in fulfilling work). Findings from the literature support the premise that engagement with alternative sources of reinforcement is negatively associated with substance use in observational and experimental studies (e.g., [1,2,3,4]), as well as in the substance use treatment setting [5]. Conversely, high substance use has been found to be associated with restricted sources of alternative reinforcement (e.g., [1,2,6]). Recently, researchers have highlighted external contextual factors (e.g., social and environmental) as influencing both the availability and valuation of alternative substance-free sources of reinforcement, which may be important to consider in the context of substance use [7]. Overall, through this lens, substance use can be viewed as a behavioral process that may be influenced by, as well as influence, the availability of alternative sources of reinforcement in an individual’s environment, and that context and type of alternative reinforcer may be important to consider.

In the United States (US), certain substances are widely available and legal for consumption, such as alcohol and nicotine. As a result, alcohol and nicotine may be sought out as primary sources of reinforcement, particularly when alternative sources of reinforcement are limited. Overall, this premise has been supported by research from the literature on alcohol [8,9,10,11] and nicotine [12,13,14,15,16].

An additional substance that is widely available and normatively consumed among the population is hyper-palatable food (HPF), operationalized as food that contains combinations of salt, sugar, carbohydrates, and/or fat at moderate to high levels [17,18,19]. In some areas of the literature, HPF have been viewed as a substance and have been proposed to be the “drug” that contributes to addictive eating behaviors and binge eating because they are potent reinforcers [20]. Thus, binge eating-related behaviors typically involve the consumption of HPF [21,22]. Binge-eating-related behaviors may be viewed on a continuum of eating behavior, ranging from passive over consumption to loss-of-control eating. For example, passive overconsumption may occur when individuals consume more HPF than intended, either when distracted or due to the rewarding effects of HPF [23,24,25]. Individuals have also been found to engage in the excess consumption of HPF during clinically identified, objective binge eating episodes [21,22]. In parallel with the findings that greater substance use may be associated with lower reinforcement from one’s environment [8,9,10,11], individuals who engage in more binge-eating-related behavior may also experience lower reinforcement from other environmental sources. However, no prior work has directly tested the role of environmental reinforcement in binge eating.

Increasing engagement with alternative sources of reinforcement may reduce the reliance on substances like alcohol, nicotine, and HPF. This premise is well aligned with the United Nation’s Sustainable Development Goals, which include targets to reduce substance use and unhealthy eating patterns through prevention approaches [26]. However, a barrier to leveraging alternative sources of reinforcement is that it is unclear which aspects of environmental reinforcement (e.g., social or occupational) may serve as alternative sources of reinforcement to substance use. Only three studies have explicitly investigated the types of environmental reinforcement that may be associated with the degree of substance use [27,28,29]. One study focused on the co-use of alcohol and cannabis and found that greater reinforcement from social activities that did not involve substance use and school activities was associated with fewer days of co-use among college freshmen [27]. Another study focused on types of environmental reinforcement and body mass index (BMI; assumed to be a proxy for HPF use) and found that a lower BMI was associated with greater engagement in social activities (that did not involve food), physical activities (e.g., running), and cognitive-enriching behaviors (e.g., reading) [28]. Another study used an ecological momentary assessment to examine behaviors in real time and found no significant associations between types of reinforcement and odds of HPF use [29]. Overall, it remains largely unclear whether specific types of environmental reinforcement may differentially influence substance use.

It has been well documented that when access to substance-free sources of reinforcement is highly constrained, the consumption of substances increases [7,30,31,32,33,34,35]. Therefore, it can be helpful to account for circumstances that may limit access to sources of reinforcement. The COVID-19 pandemic resulted in substantial restrictions on individuals’ access to sources of environmental reinforcement through lockdowns imposed early on in the pandemic (i.e., Spring 2020), when stay-at-home orders were issued in most states. Specifically, the first stay-at-home order in the US was issued by California on 19 March 2020, and by the end of May 2020, most US states and territories had issued their own stay-at-home orders [36]. These stay-at-home orders reduced the availability of sources of reinforcement in an unprecedented manner for the population [31]. During this period, access to substances was not restricted, and in some cases was enhanced due to the addition of delivery services for substances including HPF and alcohol. Accordingly, the use of alcohol [37], nicotine [38], and binge eating increased during this time [39]. Thus, the COVID-19 pandemic may have limited individuals’ engagement in reinforcing activities that do not involve substance use in unique ways that could differ from individuals before the pandemic. For example, lockdowns enforced early on in the pandemic (i.e., Spring 2020) imposed restrictions on social (e.g., going to a party), work (e.g., going to the office), leisure (e.g., going to a concert), and other activities.

The purpose of the current study was twofold. First, the study tested the associations between different sources of environmental reinforcement (e.g., social and work) and engagement in alcohol use, nicotine use, and binge eating in a large, cross-sectional sample of adults from the US. We also tested whether there were differences in the association between sources of environmental reinforcement and risky behaviors among individuals before COVID-19 lockdown/stay-at-home orders (February 2020) compared to those during COVID-19 stay-at-home orders (May 2020).

## 2. Methods

### 2.1. Study Overview, Recruitment, and Eligibility

Data were obtained from a cross-sectional survey study that examined sources of environmental reinforcement, decision making, and substance use among US adults. The study was conducted in Spring 2020 and was approved by the governing Institutional Review Board. Participants were recruited and screened using Amazon Mechanical Turk (MTurk), a crowdsourcing platform that has demonstrated reliability and validity when assessing substance use-related constructs [40]. Eligible individuals provided informed consent and completed questionnaires for 45–60 min that assessed (1) demographic information; (2) environmental reinforcement; (3) alcohol consumption; (4) nicotine use; and (5) binge eating.

Eligibility criteria were broad to facilitate the recruitment of a general sample of US-dwelling adults: (1) aged 18–65 years old; (2) located in the US; and (3) ≥99% approval rating and completed >1000 studies on MTurk to increase the likelihood of obtaining high-quality study data [41]. Surveys were available at various times and days of the week to facilitate the recruitment of participants with varied schedules.

An initial sample of 602 adults completed the study with approximately half (*n* = 304) recruited in early to mid-March of 2020, before pandemic stay-at-home orders were broadly enacted [36]. The remaining participants (*n* = 298) were recruited from early to mid-June 2020, providing data for the period when pandemic stay-at-home orders were in place for most of the US [36]. Therefore, participants in March 2020 were considered as the pre-COVID-19 group, and participants in June 2020 were considered to be the During-COVID-19 group. For questionnaires assessing behaviors over the past 30 days, participants recruited in March reported on February 2020, representing a period before COVID-19 pandemic restrictions were broadly enacted (Pre-COVID-19 period), and participants recruited in June reported on May 2020, representing a period during which COVID-19 stay-at-home orders were largely in place (During-COVID-19 period).

Within the initial sample, six individuals provided poor-quality data (i.e., missed at least two out of three attention checks) and were excluded from analyses. The final study sample was *N* = 596, consisting of 300 Pre-COVID-19 participants and 296 During-COVID-19 participants. The resulting sample size was acceptable to conduct all planned analyses (see Section 2.3 Data Analysis) [42,43].

### 2.2. Measures

#### 2.2.1. Demographics

Participants reported the following demographic information: age, race, ethnicity, and gender. For gender, participants were provided six options to choose from, which included: male; female; transgender, male to female; transgender, female to male; intersex; and prefer not to answer. All participants selected either male or female response options. Therefore, gender was transformed into a dichotomous variable for analyses.

#### 2.2.2. Environmental Reinforcement

The Pleasant Events Schedule (PES) [44] is a 320-item self-report questionnaire designed to measure the positive reinforcement an individual derived from various activities over the past 30 days. Individuals responded to items assessing both the frequency of engagement and subjective enjoyment of each activity. Frequency ratings ranged from 0 (never happened over the past month), 1 (happened a few times—1 to 6 times), to 2 (happened often—7 or more times). Enjoyment ratings were assessed on a 5-point scale ranging from not pleasant to extremely pleasant. The measure of reinforcement derived from each activity was calculated as the product of each item’s frequency and enjoyment scores. The questionnaire has previously demonstrated acceptable reliability as well as good predictive, concurrent, and construct validity within various contexts (e.g., depression and substance use) [44].

A modified PES consisting of 39 items was used in the current study. No published literature has assessed the factor structure of the PES, but researchers have modified and adapted it to use a smaller subset of items targeting their areas of interest, reporting good internal consistency following such modifications [15,34]. Due to the lack of an established factor structure, items were selected a priori based on their likely alignment with activity groupings most relevant to the current study (e.g., school/work, family, and leisure). To capitalize on the strength of the PES not being substance-specific, substance-related activity items were not excluded, allowing for the ability to examine reinforcement across multiple substances.

The measure’s assessment of frequency and enjoyment was particularly useful in examining potential differences across Pre- and During-COVID-19 time periods. Individuals who rated an activity highly enjoyable but did not engage in the activity in the past 30 days would have received a lower score than those who had high enjoyment and activity frequency ratings.

#### 2.2.3. Alcohol Use

The Alcohol Use Disorder Identification Test (AUDIT) [45] is a 10-item self-report questionnaire designed to measure alcohol consumption, including both hazardous use and related problems. Total scores ranged from 0 to 40. In the current study, the AUDIT timeframe was modified to assess the past 30 days. The AUDIT has strong psychometric properties [46] and has been validated for use in adult community samples, including those recruited using MTurk [40]. The current study yielded a McDonald’s omega of 0.86, indicating good internal consistency [47].

#### 2.2.4. Nicotine Use

The Vaping and Smoking Questionnaire from the International Tobacco Control (ITC) Policy Evaluation Project was adapted for the study [48]. Eight items assessed type of nicotine product use and frequency of use. Individuals were grouped for analysis based on their frequency of use of cigarettes and/or e-cigarettes (i.e., not at all, occasional, monthly, weekly, and daily). Among those who endorsed the dual use of tobacco cigarettes and e-cigarettes, the higher frequency rating was used to group the participants for analysis.

#### 2.2.5. Binge Eating

The Eating Pathology Symptom Inventory (EPSI) [49] is a 45-item self-report questionnaire designed to measure 8 areas of eating behaviors on a dimensional scale over the past 30 days. The questionnaire is appropriate for use in samples with and without eating disorders. The binge eating subscale, which has eight items assessing both overeating and loss-of-control eating [49], was used in the current study. As a standalone subscale, the binge eating subscale has demonstrated strong discriminant, criterion, and convergent validity among non-clinical community samples comprising adults [50,51]. The binge eating scale for the current study had a McDonald’s omega of 0.91, indicating excellent internal consistency [47].

### 2.3. Data Analysis

Data were prepared for analysis using R version 4.1.2 [52]. Sum scores were calculated for the AUDIT and the EPSI binge eating subscale, and current nicotine use frequency was coded on a 5-point scale (i.e., 0 = not at all; 1 = occasional; 2 = monthly; 3 = weekly; 4 = daily). Reinforcement from each PES item was calculated by multiplying its corresponding frequency and enjoyment scores. Independent sample *t*-tests were used for continuous variables and *χ*^2^ tests were used for categorical variables to examine potential demographic differences across Pre- and During-COVID-19 groups.

Data were analyzed using Mplus version 8.1 [53]. For the analyses, we followed a multi-step process recommended by Morin [54]. To achieve the ultimate goal of regressing our outcomes (i.e., AUDIT, EPSI, and nicotine use) on latent variables/factors underlying the PES reinforcement scores and comparing these results across cross-sectional periods (i.e., Pre-COVID-19 and During-COVID-19 groups), several data preparation requirements were addressed first, as described below.

First, we excluded PES items for which >100 participants had missing data (8 items total, see Supplementary Materials). The rationale for this choice was that many items were irrelevant for many participants (e.g., “Being with my grandchildren”), and the goal was to capture more general/broadly applicable aspects of reinforcement.

Second, as a part of basic specification, we determined how many correlated factors to extract from the PES items. We fit exploratory factor analysis (EFA) models with between 1 and 10 correlated factors; these models were estimated using the robust maximum likelihood (MLR) method [55] and Geomin rotation. Missing item-level data were accommodated using the full information maximum likelihood (FIML) method. Two complementary approaches were used to arbitrate between the EFA models with different numbers of factors. Parallel analysis was used to compare the extracted eigenvalues to those of simulated data [56], and the theoretical coherence of the models suggested by parallel analysis was also compared to determine the final number of factors to extract [57].

Third, the selected measurement model was tested to determine which parameters were invariant (i.e., equivalent) across study periods (Pre-COVID-19 and During-COVID-19 periods). This step was crucial as the regression pathways between study periods should only be compared if certain parameters are invariant. Our preferred EFA measurement model was refit within the exploratory structural equation modeling (ESEM) [58] framework, and its invariance was tested hierarchically [59], as described in detail in the Supplementary Materials. This procedure resulted in the identification of the most invariant version of the measurement model (i.e., only allowing the minimum number of parameters to differ between the Pre-COVID-19 and During-COVID-19 periods).

Finally, the hypothesized regression pathways (and the covariate of gender) were added to this version of the model, and its structural invariance was tested hierarchically (see Supplementary Materials). The parameters of this model answered our questions about the relationships between the PES factors and the outcome variables, and the results of the structural invariance testing answered our questions about the impact of the COVID-19 pandemic on these relationships. If the fit did not significantly worsen by the fourth and final model, this indicated that predictive invariance was supported and that the relationship between the ESEM factors and outcomes was the same for both study periods. If the fit did significantly worsen at any level, this would have indicated the effect of COVID-19 on said relationship.

## 3. Results

### 3.1. Descriptive Statistics

Participant characteristics and descriptive statistics for outcome measures are presented in Table 1. Approximately half of the participants (53.2%) identified as male and 72.7% identified as White, non-Hispanic. Across the Pre-COVID-19 and During-COVID-19 study periods, there were no significant differences in participant characteristics except for gender; participants were significantly more likely to be male in the During-COVID-19 period (*χ*^2^ [1, *N* = 596] = 3.94, *p* = 0.047).

### 3.2. Basic Specification

Parallel analysis suggested that the optimal number of factors was approximately five; therefore, we considered models with 4–6 factors (see Supplemental Figure S1). All three models had an acceptable model fit (i.e., CFI > 0.90, RMSEA < 0.05, and an SRMR < 0.05; see Supplemental Table S1). When examining the factor loadings, the four-factor model appeared to be the most conceptually cohesive model that also demonstrated an acceptable model fit (see Supplementary Materials). This model was retained when looking at the Pre-COVID-19 period and During-COVID-19 period individually as well (see Supplemental Table S1). A table with the standardized factor loadings for the four-factor model is presented in Supplemental Table S2.

### 3.3. Measurement Invariance

A hierarchical process was used to assess measurement invariance for the four-factor ESEM model across study periods and to identify a version of the model that met partial strict invariance (Table 2; further detailed in the Supplementary Materials). The resulting partial strict invariance model was comparable in fit to the strong invariance model (∆CFI = −0.005, ∆TLI = −0.001, ∆RMSEA = 0, and ∆SRMR = +0.006). A table with the standardized factor loadings for the partial strict invariance model is presented in Table 3. The model included four factors, with strong standardized factor loadings representing social-related activities (e.g., activities with friends; social gatherings), romantic-related activities (activities with an intimate partner), work/school-related activities (activities at work, most of which included social-based activities within the work/school environment), and daily living activities.

### 3.4. Predicitve Models

**Figure 1:**
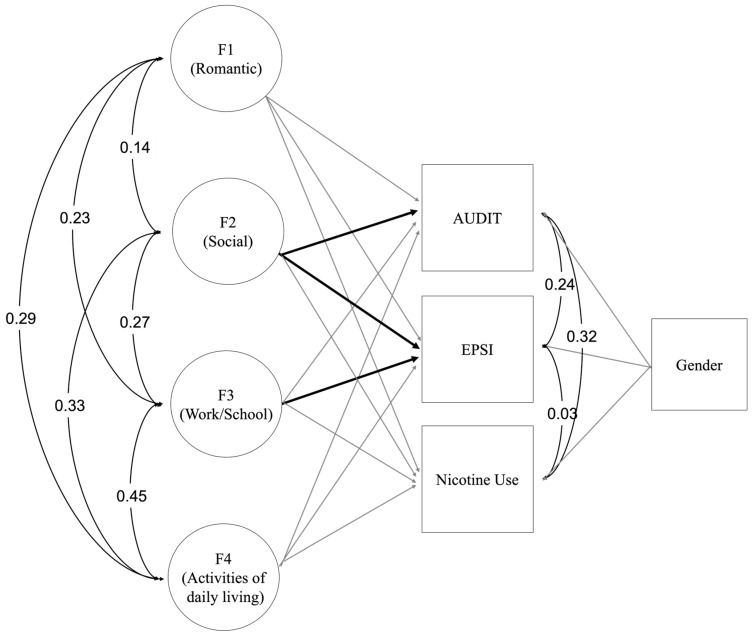
Partial strict invariance 4-factor predictive model (slopes, intercepts, and residual variances constrained across timepoints). *Note*: Statistically significant regression pathways are black and non-significant regression pathways are gray. Outcome and predictor correlations are indicated in the figure. The area of activities that each factors strong loadings are most related to are included in parentheses.

The four-factor ESEM model for the partial strict invariance solution was adjusted to generate a predictive model. First, we estimated all predictions freely, allowing for regression pathways to be different across COVID-19 periods (see Supplemental Table S3a,b). An interaction effect of COVID-19 period was assessed and, as more constraints were added at each step, the fit of the model either stayed the same or minimally diminished, maintaining a satisfactory fit to the data (see Table 2 for change in fit indices). When the interaction effect of COVID-19 period was included, a review of model fit indices indicated that the model fit did not substantially diminish (none of the nested models exceeded a change of −0.01 in both the CFI or TLI, nor was there a change of +0.015 in the RMSEA or SRMR). This was the case for all models, including the more constrained models and the final model that had slopes, intercepts, and residual variances constrained to equality (∆ CFI = −0.001, ∆ TLI = 0, ∆ RMSEA = 0, and ∆ SRMR = 0). Given the model fit did not substantially worsen, as more components of the predictive model were constrained, this indicated that the model functioned similarly across periods and that there was no real interaction effect of COVID-19 period. Following this process, the most constrained predictive model was used for further interpretation across groups. The structural model is presented in Figure 1, and results with standardized parameter estimates are presented in Table 4.

The results indicate that social-related environmental reinforcement was significantly positively associated with both alcohol consumption (*β* = 0.30, *p* < 0.001) and binge eating behaviors (*β* = 0.26, *p* < 0.001). For every standard deviation increase in the social-related environmental reinforcement factor, alcohol use and related problems were 0.30 standard deviations higher, and binge eating behaviors were 0.26 standard deviations higher on average. The results also indicate that work/school-related environmental reinforcement was significantly negatively associated with binge eating behaviors (*β* = −0.14, *p* = 0.014). For every standard deviation increase in the work/school-related environmental reinforcement factor, binge eating behaviors were 0.14 standard deviations lower on average. The work/school-related environmental reinforcement factor was not significantly associated with alcohol use and related problems (Table 4). None of the environmental reinforcement factors was significantly associated with nicotine use (Table 4).

## 4. Discussion

The current study examined whether different types of reinforcement from the environment (e.g., social or work) were associated with multiple health risk behaviors (i.e., alcohol use, binge eating, and nicotine use) among a general community sample of US adults. The study first explored and tested the factor structure of the Pleasant Events Schedule (PES), a commonly used measure to assess sources of environmental reinforcement, and then used the resulting factor structure to test associations between types of environmental reinforcement and multiple risky behaviors. The results indicate a four-factor structure for the PES demonstrates the most conceptually cohesive model and acceptable model fit to address the study’s aims. Furthermore, evidence for predictive invariance indicates the structure of the model functioned similarly across samples from Pre-COVID-19 and During-COVID-19 periods, indicating that the relationship between the PES factors and outcome variables are the same for both periods. Thus, when testing the association between areas of reinforcement and health risk behaviors, greater social-related reinforcement was associated with more alcohol use and binge eating behavior, and greater work/school-related reinforcement was associated with lower engagement in binge eating behavior. No areas of environmental reinforcement were significantly associated with current nicotine use. Collectively, the findings identify social-related and work/school-related sources of environmental reinforcement as having nuanced associations with alcohol use and binge eating behavior among a general sample of adults.

The current study findings reveal a positive relationship between social-related reinforcement and alcohol use and binge eating. The assessment of reinforcement from social activities included activities that could involve substance use. Our findings that social-related activities (that may involve substance use) were positively associated with alcohol use are consistent with prior literature documenting the positive association of alcohol use and related problems with greater social involvement, attendance at parties, and related social gatherings [60,61,62,63]. However, the finding regarding social reinforcement and binge eating is unique and suggests that binge eating may function similarly in social settings as alcohol use. Given the extensive prevalence of HPF in the US food environment (69% of available foods as of 2018 [19]) and the substantial degree to which socialization and food intake co-occur in US society [64,65,66], this finding may have broad generalizability to the US population.

The results of the current study also suggest that work/school-related reinforcement may be associated with lower binge eating behavior. This finding is new and possibly important as it may suggest that reinforcement from work/school activities may provide a buffer against engagement in binge eating behavior. The work/school factor in the study consisted mostly of social-based activities within the work/school environment; therefore, it is possible that the observed negative association may be explained by engaging in social-related reinforcing activities at work/school, which may promote less binge eating. In the prior literature, work/school-related activities have been found to increase stress [67,68,69], which has been found to be a trigger for binge eating behaviors [70]. Therefore, it is interesting that our findings suggest that work/school-related reinforcement, most of which was social-related, may be associated with reduced binge eating behavior. Another possible explanation for the observed negative association between work/school sources of reinforcement and binge eating may be that individuals had a lack of opportunity to engage in binge eating behaviors in the work/school setting (e.g., access to food and time). However, it is worth noting that this finding was present even for those assessed during the early lockdown periods during the COVID-19 pandemic, when most people were at home for extended periods of time and may have had more opportunities to engage in binge eating behavior. Overall, considering our findings within the context of the literature, it may be that work/school-related sources of reinforcement that are primarily social in nature may be associated with less binge eating, whereas stress from work/school can be a trigger for binge eating behavior. Future research should focus on replicating these findings and further delineating the characteristics of the work/school environment that could be associated with lower or higher binge eating behavior, which may have clinical utility.

The findings indicate that no areas of environmental reinforcement were significantly associated with current nicotine use. The existing literature largely supports the premise that environmental reinforcement may yield lower rates of nicotine use and help sustain less use over time [12,13,14,15,16]. Our null findings may be due to a relatively small proportion of the sample endorsing current nicotine use (21.6%), with most endorsing daily use (16.8%). Additionally, the current study combined individuals who endorsed current cigarette and e-cigarette use into a single frequency variable, possibly impacting the resolution to detect associations across individuals who used cigarettes and/or e-cigarettes at varying frequencies. Further work is needed to investigate the degree to which environmental reinforcement may be negatively associated with nicotine use.

The current study applied a novel analytic methodology using ESEM and is, to our knowledge, the first to rigorously examine the factor structure of the PES. Specifically, despite its wide use in the literature, researchers have not conducted an analysis of the PES factor structure, which is a standard method to evaluate the item structure and psychometric properties of a measure [71,72]. Instead, researchers have typically grouped PES items together and calculated summary scores without an evaluation of the measure’s factor structure to inform the item groupings (e.g., [15,34]). This approach may limit researchers’ ability to draw accurate conclusions about associations between environmental reinforcement and substance use, as groupings of items may not align with the reality of how the activities function for individuals [73]. Thus, our approach employed ESEM to evaluate the factor structure of the PES using data-informed item groupings, and demonstrated utility in assessing environmental reinforcement in a manner that facilitated analysis with multiple risky behaviors. The findings regarding a possible factor structure for the PES may be useful to other researchers interested in examining environmental reinforcement with greater specificity for the types of reinforcing activities and better psychometric support. Since the PES does not differentiate substance-involved from substance-free sources of reinforcement, the measure facilitated an analysis of sources of reinforcement with multiple risk behaviors concurrently, and the patterns of our findings reveal the utility of such an approach.

### Limitations

The current study had several limitations. First, the model fit for the final model was acceptable, but not excellent. However, the primary aim was not to determine the optimal structure of the PES, but rather to find an acceptable exploratory structure that provided a more empirically driven fit to the data, an improvement over previous approaches in the literature. Second, we used a convenience sample that was composed of predominately White and non-Hispanic US adults from a crowdsourcing platform. Therefore, our findings may not be generalizable to more diverse communities represented in and outside the US [74]. This is salient as research has indicated that access to sources of alternative reinforcement may vary among populations not well represented in our study [75,76,77]. However, the study itself had a large sample size, and the findings provide initial contributions to a limited existing literature base. Finally, due to this nascent area of research and the preliminary nature of this study, more work is needed to determine if similar effects can be replicated. Until then, cautious interpretations about the current findings should be made.

## 5. Conclusions

The findings from the current study identify a possible four-factor structure for a modified version of the PES, which can be built upon in future research. Social-related environmental reinforcement was consistently associated with higher engagement in multiple health risk behaviors, suggesting that social-related reinforcement may be related to greater alcohol use and binge eating behavior. Work/school-related environmental reinforcement was negatively associated with binge eating behavior and suggested that a greater engagement in reinforcing work/school activities may be related to lower binge eating behavior. No areas of environmental reinforcement were associated with nicotine use. This study extends the current literature as the findings suggest that separating environmental reinforcement into specific areas can aid in identifying which activities may be associated with a greater or lower engagement in multiple health risk behaviors. Understanding these relationships can inform future studies testing prevention and intervention efforts to reduce engagement in health risk behaviors.

## Figures and Tables

**Table 1:** Demographic characteristics of study sample and descriptive statistics of outcome measures.

Variable	Full Sample (*N* = 596)	Pre-COVID-19 Period (*N* = 300)	During-COVID-19 Period (*N* = 296)
**Gender**			
Male	317 (53.2%)	147 (49.0%)	170 (57.4%)
Female	277 (46.5%)	152 (50.7%)	125 (42.2%)
**Race/Ethnicity**			
White/Non-Hispanic	433 (72.7%)	219 (73.0%)	214 (72.3%)
White/Hispanic	30 (5.0%)	16 (5.3%)	14 (4.7%)
Black/Non-Hispanic	47 (7.9%)	24(8.0%)	23 (7.8%)
Asian/Non-Hispanic	51 (8.6%)	23 (7.7%)	28 (9.5%)
Multiracial/Ethnicity	35 (5.9%)	18 (6.0%)	17 (5.7%)
**Age**	37.81 (SD ± 10.72)	37.41 (SD ± 10.49)	38.22 (SD ± 10.95)
**Current Nicotine Use**			
None	467 (78.4%)	229 (76.3%)	238 (80.4%)
Occasional	7 (1.2%)	2 (0.7%)	5 (1.7%)
Monthly	5 (0.8%)	1 (0.3%)	4 (1.4%)
Weekly	17 (2.9%)	8 (2.7%)	9 (3.0%)
Daily	100 (16.8%)	60 (20.0%)	40 (13.5%)
**AUDIT Total**	3.78 (SD ± 5.03)	3.93 (SD ± 4.85)	3.63 (SD ± 5.21)
**EPSI Binge Eating Subscale Total**	8.01 (SD ± 6.28)	8.64 (SD ± 6.50)	7.36 (SD ± 5.99)

*Note*. Abbreviations are SD (standard deviation); AUDIT (Alcohol Use Identification Test); EPSI (Eating Pathology Symptom Inventory).

**Table 2:** Goodness-of-fit indices associated with the measurement and predictive 4-factor invariance hierarchical models.

Invariance Models	*χ*^2^ diff (∆df)	*p*-Value	CFI	∆ CFI	TLI	∆ TLI	RMSEA (90% CI)	∆ RMSEA	SRMR	∆ SRMR
Measurement Invariance										
Configural Invariance	-	-	0.888	-	0.850	-	0.055 (0.050, 0.059)	-	0.045	-
Weak (Metric) Invariance	131.683 (108)	0.060	0.886	−0.002	0.867	0.017	0.052 (0.047, 0.056)	−0.003	0.053	0.008
Strong (Scalar) Invariance	44.157 (27)	0.020	0.883	−0.005	0.869	0.019	0.051 (0.047, 0.055)	−0.001	0.056	0.011
Strict (Residual) Invariance	102.490 (31)	<0.001	0.862	−0.024	0.851	−0.016	0.055 (0.051, 0.059)	0.004	0.075	0.022
Partial Strict (Residual) Invariance	55.935 (30)	0.003	0.878	−0.005	0.868	−0.001	0.051 (0.047, 0.056)	0.000	0.062	0.006
Predictive Invariance										
No Constraints	283.488 (218)	0.002	0.872	−0.006	0.859	−0.009	0.048 (0.044–0.052)	−0.003	0.059	−0.003
Slopes	19.628 (13)	0.105	0.871	−0.001	0.860	0.001	0.048 (0.044–0.052)	0.000	0.061	0.002
Slopes and Intercepts	5.878 (3)	0.118	0.871	0.000	0.859	−0.001	0.048 (0.044–0.052)	0.000	0.061	0.000
Slope, Intercepts, and Residual Variances	10.500 (5)	0.062	0.870	−0.001	0.859	0.000	0.048 (0.045–0.052)	0.000	0.061	0.000

*Note*. Abbreviations are *χ*^2^ diff (Satorra-Bentler scaled chi-square difference test); ∆df (difference of degrees of freedom); CFI (comparative fit index); TLI (Tucker–Lewis index); RMSEA (root mean square error of approximation); CI (confidence interval); SRMR (standardized root square mean residual); ∆ in front of the fit indices denotes the change from one invariance model from the one that comes right before it. Predictive invariance models include the final partial strict invariance model with regressions added and constraints incrementally added.

**Table 3:** Standardized factor loadings for the partial strict invariance four-factor model.

PES Items	Factor 1		Factor 2		Factor 3		Factor 4	
	P1	P2	P1	P2	P1	P2	P1	P2
Thinking about people I like (2)	0.16	0.16	0.00	0.00	0.27	0.26	0.20	0.22
Being with my significant other/spouse/partner (3)	**0.90**	**0.90**	−0.03	−0.03	0.06	0.06	−0.02	−0.02
Going to a party (4)	0.01	0.01	**0.66**	**0.63**	0.06	0.06	0.05	0.05
Meeting someone new (5)	−0.03	−0.03	**0.46**	**0.44**	0.25	0.25	0.06	0.07
Being with friends (6)	0.08	0.09	**0.47**	**0.45**	0.24	0.24	0.02	0.02
Making a new friend (7)	0.03	0.03	**0.54**	**0.52**	0.04	0.05	0.26	0.31
Seeing old friends (8)	0.00	0.00	**0.53**	**0.50**	0.06	0.06	0.15	0.18
Having company over (9)	0.12	0.13	**0.58**	**0.56**	0.04	0.04	0.12	0.14
Having a party or get-together (10)	0.09	0.10	**0.67**	**0.66**	0.04	0.04	0.16	0.19
Seeing good things happen to my family or friends (11)	0.08	0.09	0.19	0.17	0.33	0.32	0.13	0.15
Buying something for my family (12)	0.11	0.11	0.03	0.02	0.23	0.22	0.31	0.34
Being at a family event or get-together (14)	0.05	0.06	**0.60**	**0.57**	0.03	0.03	0.14	0.17
Being with my parents (15)	−0.11	−0.11	0.27	0.25	0.11	0.10	0.19	0.21
Having sexual relations with a partner (18)	**0.80**	**0.83**	0.09	0.08	0.03	0.03	0.06	0.06
Kissing (19)	**0.91**	**0.92**	0.02	0.02	0.01	0.01	0.05	0.05
Caressing a partner (20)	**0.90**	**0.91**	−0.02	−0.02	0.03	0.03	0.03	0.03
Talking about sex (21)	**0.49**	**0.50**	0.11	0.10	−0.08	−0.08	0.24	0.27
Going to work or school (22)	0.02	0.02	0.00	0.00	**0.54**	**0.53**	0.20	0.22
Talking about work or school (23)	0.02	0.02	0.20	0.18	**0.42**	**0.43**	0.26	0.31
Doing a job well (25)	0.05	0.05	−0.06	−0.05	**0.59**	**0.57**	0.11	0.12
Pleasing employers, teachers, etc. (26)	0.06	0.07	0.00	0.00	**0.67**	**0.65**	0.06	0.07
Talking with people on the job or in school (27)	0.06	0.07	0.14	0.13	**0.66**	**0.66**	0.03	0.03
Going to work parties or get-togethers (28)	0.02	0.02	**0.62**	**0.59**	0.02	0.02	0.14	0.16
Relaxing (29)	0.06	0.06	−0.14	−0.12	0.11	0.10	0.19	0.21
Reading (30)	0.05	0.05	−0.15	−0.13	0.23	0.22	0.15	0.17
Biking (32)	0.02	0.02	0.28	0.25	−0.12	−0.12	0.25	0.27
Going to a movie (34)	0.01	0.02	0.39	**0.63**	0.10	0.18	0.05	0.09
Doing housework or laundry; cleaning things (36)	0.01	0.01	0.03	0.03	0.09	0.08	0.33	0.37
Writing emails, texts, or letters (37)	0.02	0.02	−0.07	−0.06	0.04	0.04	**0.71**	**0.73**
Getting emails, texts, or letters (38)	0.02	0.02	−0.04	−0.04	0.01	0.01	**0.71**	**0.74**
Taking a walk (39)	0.06	0.07	0.08	0.08	0.11	0.10	0.35	0.38

*Note*. Items 1, 13, 16, 17, 24, 31, 33, and 35 were all removed due to missingness >99; strong loadings (>0.40) are bolded. Item 34 allowed us to differ between periods. P1 = Pre-COVID-19 period. P2 = During-COVID-19 period. Strong standardized factor loadings for Factor 1 = romantic-related activities, Factor 2 = social-related activities, Factor 3 = work/school-related activities, and Factor 4 = activities related to daily living. Given that Factor 4 only has two strong indicators, this factor may be less replicable.

**Table 4:** Four-factor latent regression ESEM model (partial strict invariance and full predictive invariance).

Predictor → Outcome	Standardized Estimate		95% CI			*p*-Value
			Lower	Upper		
F1 → AUDIT	−0.05		−0.13	0.04		0.259
F2 → AUDIT	0.30		0.18	0.41		<0.001
F3 → AUDIT	−0.10		−0.21	0.02		0.089
F4 → AUDIT	−0.02		−0.13	0.10		0.769
Female → AUDIT	−0.07		−0.15	0.01		0.074
						
F1 → EPSI	0.00		−0.09	0.09		0.969
F2 → EPSI	0.26		0.15	0.36		<0.001
F3 → EPSI	-0.14		−0.26	−0.03		0.014
F4 → EPSI	0.01		−0.10	0.12		0.865
Female → EPSI	0.04		−0.04	0.12		0.368
						
F1 → Nicotine Use	−0.01		−0.10	0.07		0.755
F2 → Nicotine Use	0.02		−0.08	0.12		0.678
F3 → Nicotine Use	0.11		−0.01	0.22		0.069
F4 → Nicotine Use	0.04		−0.08	0.15		0.539
Female → Nicotine Use	0.04		−0.04	0.13		0.330

*Note*. Standardized estimates are Beta coefficients for all outcome variables. Abbreviations are Predictor → Outcome (outcome variable regressed on predictor variable); 95% CI (95% confidence interval of the standardized estimate); Lower (lower-limit of the 95% CI); Upper (upper-limit of the 95% CI); F1 (romantic-related activities factor); F2 (social-related activities factor); F3 (work/school-related activities factor); F4 (activities related to daily living factor); AUDIT (Alcohol Use Identification Test); EPSI (Eating Pathology Symptom Inventory).

## Data Availability

All data, analysis code, and research materials are available by contacting the corresponding authors and with IRB approval. The data are not publicly available due to privacy and ethical restrictions.

## Supplementary Materials

The following supporting information can be downloaded at: https://www.mdpi.com/article/10.3390/ijerph21111390/s1, Supplementary Materials for the methods and results; Figure S1: Parallel analysis-produced scree plot; Table S1. Initial fit indices for suggested factors to retain from the parallel analysis; Table S2. Standardized factor loadings for the full sample four-factor model; Table S3a. Four-factor latent regression ESEM model (partial strict invariance—Pre-COVID-19 period); Table S3b. Four-factor latent regression ESEM model (partial strict invariance—during-COVID-19 period). Reference [78] is cited in the Supplementaty Materials.
