# Supplementary material for: Sources of Environmental Reinforcement and Engagement in Health Risk Behaviors Among a General Population Sample of US Adults
Source: Int J Environ Res Public Health. 2024 Oct 22;21(11):1390. doi: 10.3390/ijerph21111390 (PMC11593772; doi:10.3390/ijerph21111390)
Supplement: Supplementary file 1 [file ijerph-21-01390-s001.zip › ijerph-3138122-supplementary.pdf]

Supplemental Information

Journal: *International Journal of Environmental Research and Public Health*

Manuscript Title: Sources of Environmental Reinforcement and Engagement in Health Risk Behaviors Among a General Population Sample of US Adults

Authors: Alexa M. L'Insalata., Jeffrey M. Girard., and Tera L. Fazzino

2. Methods

2.3. Data Analysis

Basic Specification. The first step of the parallel analysis included constructing 50 random correlation matrixes to match the real dataset in terms of the sample size and number of variables and extracting their eigenvalues using principal components analysis [56]. Then both the average and 95th percentile of all generated eigenvalues from the random correlation matrixes were plotted with the extracted eigenvalues for the current study data on a scree plot as the final output to be evaluated [56]. The factors retained for a further evaluation of their overall model fit statistics included those whose sample eigenvalues overlapped with, but remained greater than, those of the random data. As Morin [54] recommended, the first step before delving into assessing the ESEM model fit and subsequent measurement invariance is fitting a CFA model and comparing it to the ESEM model to demonstrate the evidence of using ESEM over CFA. In our case, the items that make up the modified Pleasant Event Schedule (PES) we used do not have a supported a priori factor structure. This is largely our reasoning for using ESEM to avoid inappropriately grouping items we thought might fit together for analyses post hoc. Given this, it was reasonable to not include this step as part of the data analyses.

Items that had 100 or more observations missing and were removed from the factor analysis included the following eight items: "Dating" (1); "Talking about my children or grandchildren" (13); "Being with my children" (16); "Being with my grandchildren" (17); "Getting a job advancement (being promoted, given a raise-or offered a better job, etc.)" (24); "Playing a musical instrument" (31); "Playing recreational sports" (33); "Going to a play" (35).

Measurement Invariance. There are four levels of measurement invariance: *configural invariance* requires the model structure (i.e., which items load on which factors) to be equivalent across samples; *weak invariance* also requires all factor loadings to be equivalent; *strong invariance* also requires all item intercepts and thresholds to be equivalent; and finally, *strict invariance* also requires all residual variances to be equivalent [53]. Models were fit to our data that imposed the constraints of each level of invariance, and each model was compared to the model of the previous level [78]. A decrease in the CFI and/or TLI ≥ 0.01 and an increase in the RMSEA of ≤ 0.015 was taken to indicate that the less constrained model should be retained and invariance was not achieved. In practice, it is often useful to attain "partial" invariance if a level of invariance cannot be "fully" achieved [78]. For example, a model that has strong invariance but not strict invariance may be modified (e.g., by freeing a subset of residual variances) to achieve "partial" strict invariance.

Predictive Models. In the first model, all slopes and intercepts were estimated freely in both periods; in the second model, we constrained the slopes to equality; in the third model, we also constrained the intercepts to equality; and in the fourth model, we also constrained the disturbances to equality (the residual variances of each outcome variable). Similar to the hierarchical model for measurement invariance, changes in the model fit were assessed using the same cutoffs for the fit indices to determine structural invariance at each step.

3. Results

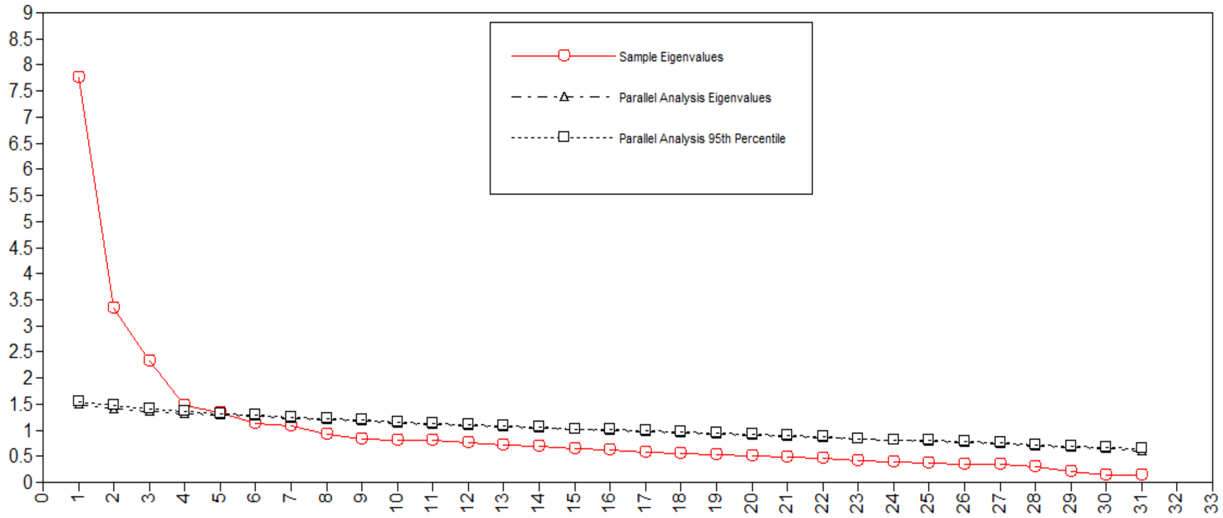

Supplemental Figure S1: Parallel analysis-produced scree plot.

Supplemental Table S1. Initial fit indices for suggested factors to retain from the parallel analysis.

Model	χ2 (df)	CFI	TLI	RMSEA (90% CI)	SRMR
6-Factor Full Sample	559.181 (294)	0.951	0.922	0.039 (0.034-0.044)	0.026
5-Factor Full Sample	650.687 (320)	0.939	0.911	0.042 (0.037-0.046)	0.031
4-Factor Full Sample	799.736 (347)	0.916	0.888	0.047 (0.043-0.051)	0.038
4-Factor Period 1 Only	674.598 (347)	0.894	0.858	0.056 (0.050-0.062)	0.043
4-Factor Period 2 Only	639.967 (347)	0.880	0.840	0.054 (0.047-0.060)	0.046

Note. Abbreviations are χ2 (chi-squared test of model fit); df (degree of freedom); CFI (comparative fit index); TLI (Tucker–Lewis index); RMSEA (root mean square error of approximation); CI (confidence interval); SRMR (standardized root square mean residual). Period 1 (Pre-COVID-19 period); Period 2 (During-COVID-19 period). The χ2Test of model fit was significant at $p < 0.001$ for all models.

Supplemental Table S2. Standardized factor loadings for the full sample four-factor model.

PES Items	Factor 1	Factor 2	Factor 3	Factor 4
Thinking about people I like (2)	−0.02	0.15	0.28	0.21
Being with my significant other/spouse/partner (3)	−0.03	**0.90**	0.06	−0.01
Going to a party (4)	**0.67**	0.01	0.10	−0.01
Meeting someone new (5)	**0.44**	-0.03	0.28	0.02
Being with friends (6)	**0.46**	0.07	0.23	0.02
Making a new friend (7)	**0.54**	0.02	0.08	0.22
Seeing old friends (8)	**0.53**	-0.01	0.09	0.12
Having company over (9)	**0.60**	0.11	0.05	0.10
Having a party or get-together (10)	**0.69**	0.08	0.06	0.11
Seeing good things happen to my family or friends (11)	0.17	0.08	0.34	0.12
Buying something for my family (12)	0.01	0.11	0.25	0.30
Being at a family event or get-together (14)	**0.63**	0.05	0.06	0.08
Being with my parents (15)	0.27	−0.11	0.13	0.16

Having sexual relations with a partner (18)	0.07	**0.81**	0.05	0.05
Kissing (19)	0.02	**0.91**	0.02	0.06
Caressing a partner (20)	−0.03	**0.91**	0.04	0.04
Talking about sex (21)	0.10	**0.49**	−0.07	0.25
Going to work or school (22)	−0.04	0.02	**0.56**	0.19
Talking about work or school (23)	0.16	0.01	**0.46**	0.25
Doing a job well (25)	−0.10	0.05	**0.61**	0.10
Pleasing employers, teachers, etc. (26)	−0.04	0.06	**0.69**	0.04
Talking with people on the job or in school (27)	0.09	0.06	**0.67**	0.03
Going to work parties or get-togethers (28)	**0.62**	0.01	0.07	0.08
Relaxing (29)	−0.15	0.06	0.12	0.20
Reading (30)	−0.15	0.05	0.24	0.14
Biking (32)	0.28	0.02	-0.07	0.19
Going to a movie (34)	**0.48**	0.04	0.11	0.07
Doing housework or laundry; cleaning things (36)	0.01	0.01	0.09	0.34
Writing emails, texts, or letters (37)	−0.08	0.01	0.01	**0.76**
Getting emails, texts, or letters (38)	−0.06	0.01	−0.04	**0.79**
Taking a walk (39)	0.08	0.06	0.13	0.34

Note: Items 1, 13, 16, 17, 24, 31, 33, and 35 were all removed due to missingness > 99; strong loadings (> 0.40) are bolded. Strong standardized factor loadings for Factor 1 = social-related activities, Factor 2 = romantic-related activities, Factor 3 = work/school-related activities, and Factor 4 = activities related to daily living.

Strong standardized factor loadings for Factor 1 ranged from 0.44 to 0.69 and represented social-related activities. Factor 2 had a range of strong factor loadings ranging from 0.49 to 0.91 and represented romantic-related activities. Factor loadings ranged from 0.46 to 0.69 on factor 3, representing work/school-related activities. The standardized factor loadings for Factor 4 had only two strong loadings greater than 0.40, indicating activities related to daily living. Given that the fourth factor only had two strong indicators, this factor may be less replicable.

3.3. Measurement Invariance

The configural model demonstrated a mediocre fit. However, fit improved across indices, except the CFI, which decreased minimally but did not exceed a change of -0.01 (Δ CFI = -0.002), when factor loadings were set to be equivalent to assess for weak invariance (see Table 2 in the manuscript for the full report of indices). This pattern of changes in fit persisted when testing for strong invariance. However, when testing for strict invariance, the model exceeded a change of -0.01 in both the CFI or TLI and a change of +.015 in the SRMR but not the RMSEA (Δ CFI = -.024, Δ TLI = -0.016, Δ RMSEA = +0.004, and Δ SRMR = +0.022). Thus, this indicated a substantial decrease in the model fit from the strong invariance model and therefore a lack of residual variance invariance. The evaluation of modification indices indicated that the failed full strict invariance model was primarily the result of item 34 "Going to the movies", as its residual variance was associated with the largest modification index (76.722). Consequently, the residual variance for item 34 was freed across COVID-19 periods.

Supplemental Table S3a. Four-factor latent regression ESEM model (partial strict invariance—Pre-COVID-19 period).

Predictor → Outcome	Standardized Estimate	95% CI		*p*-value
		Lower	Upper	
F1 → AUDIT	−0.10	−0.21	0.01	0.078
F2 → AUDIT	0.35	0.18	0.52	< 0.001
F3 → AUDIT	−0.06	−0.23	0.12	0.530

F4 → AUDIT	−0.09	−0.27	0.09	0.340
Female → AUDIT	−0.13	−0.24	−0.02	0.024
F1 → EPSI	0.04	−0.09	0.17	0.512
F2 → EPSI	0.21	0.06	0.37	0.007
F3 → EPSI	−0.32	−0.48	-0.16	< 0.001
F4 → EPSI	0.07	−0.13	0.27	0.477
Female → EPSI	0.02	−0.10	0.13	0.804
F1 → Nicotine Use	−0.10	−0.22	0.02	0.113
F2 → Nicotine Use	−0.05	−0.20	0.10	0.506
F3 → Nicotine Use	0.21	0.05	0.37	0.010
F4 → Nicotine Use	−0.02	−0.22	0.18	0.836
Female → Nicotine Use	0.02	−0.11	0.14	0.797

Note: Standardized estimates are Beta coefficients for all outcome variables. Abbreviations are Predictor → Outcome (outcome variable regressed on predictor variable); 95% CI (95% confidence interval of the standardized estimate); Lower (lower-limit of the 95% CI); Upper (upper-limit of the 95% CI); F1 (romantic-related activities factor); F2 (social-related activities factor); F3 (work/school-related activities factor); F4 (activities related to daily living factor); AUDIT (Alcohol Use Identification Test); EPSI (Eating Pathology Symptom Inventory).

Supplemental Table S3b. Four-factor latent regression ESEM model (partial strict invariance—During-COVID-19 period).

**Predictor → Outcome**	**Standardized Estimate**	**95% CI**		***p*-value**
		**Lower**	**Upper**	
F1 → AUDIT	−0.01	−0.13	0.11	0.862
F2 → AUDIT	0.24	0.07	0.41	0.005
F3 → AUDIT	−0.10	−0.24	0.03	0.122
F4 → AUDIT	0.00	−0.15	0.15	0.987
Female → AUDIT	0.00	−0.12	0.12	0.065
F1 → EPSI	−0.03	−0.16	0.09	0.605
F2 → EPSI	0.28	0.11	0.44	0.001
F3 → EPSI	0.00	−0.16	0.16	0.985
F4 → EPSI	0.02	−0.14	0.19	0.795
Female → EPSI	0.03	−0.09	0.14	0.619
F1 → Nicotine Use	0.08	−0.05	0.21	0.231
F2 → Nicotine Use	0.08	−0.07	0.23	0.301
F3 → Nicotine Use	0.00	−0.16	0.15	0.983
F4 → Nicotine Use	0.13	−0.01	0.27	0.065
Female → Nicotine Use	0.05	−0.07	0.18	0.402

Note: Standardized estimates are Beta coefficients for all outcome variables. Abbreviations are Predictor → Outcome (outcome variable regressed on predictor variable); 95% CI (95% confidence interval of the standardized estimate); Lower (lower-limit of the 95% CI); Upper (upper-limit of the 95% CI); F1 (romantic-related activities factor); F2 (social-related activities factor); F3 (work/school-related activities factor); F4 (activities related to daily living factor); AUDIT (Alcohol Use Identification Test); EPSI (Eating Pathology Symptom Inventory).

References

53. Muthén, L.K.; Muthén, B.O. *Mplus User's Guide*; Eighth.; Los Angeles, CA, 2018;
54. Morin, A.J.S. Exploratory Structural Equation Modeling. In *Handbook of Structural Equation Modeling*; Hoyle, R.H., Ed.; Guilford: New York, NY, USA, 2021; ISBN 2013206534.

56. Hayton, J.; Allen, D.; Scarpello, V. Factor Retention Decisions in Exploratory Factor Analysis: A Tutorial on Parallel Analysis. *Organ. Res. Methods* **2004**, *7*, 191–205. https://doi.org/10.1177/1094428104263675.
78. Putnick, D.L.; Bornstein, M.H. Measurement Invariance Conventions and Reporting: The State of the Art and Future Directions for Psychological Research. *Dev. Rev.* **2016**, *41*, 71–90. https://doi.org/10.1016/J.DR.2016.06.004.
